# Extremophile-Derived Bioactives in Cosmeceuticals: Bridging Nutraceuticals and Skincare for Holistic Wellness

**DOI:** 10.3390/life15121787

**Published:** 2025-11-21

**Authors:** Emanuela Maresca, Micaela Carbone, Giovanni Gallo, Salvatore Fusco, Martina Aulitto

**Affiliations:** 1 Stazione Zoologica Anton Dohrn, Department of Ecosustainable Marine Biotechnology, 80133 Napoli, Italy; emanuela.maresca@szn.it; 2 Department of Medical Sciences, University of Turin, 10126 Turin, Italy; micaela.carbone@unito.it; 3 Faculty of Biology, Microbiology, Ludwig-Maximilians-Universität München, 82152 Martinsried, Germany; 4 Biochemistry and Industrial Biotechnology (BIB) Laboratory, Department of Biotechnology, University of Verona, 37134 Verona, Italy; salvatore.fusco@univr.it; 5 Department of Biology, University of Napoli Federico II, Complesso Universitario Monte Sant’Angelo, 80126 Napoli, Italy; martina.aulitto@unina.it

**Keywords:** extremophiles, bioactive compounds, cosmeceuticals, antioxidant, sustainable biotechnology, *ectoine*, extremozymes, carotenoids, nutricosmetics

## Abstract

The integration of extremophile-derived bioactives into cosmeceuticals and nutricosmetics offers a novel strategy to enhance skin health through both topical and systemic approaches. Extremophile microorganisms, adapted to extreme conditions, produce unique compounds such as *ectoine*, extremozymes, carotenoids, exopolysaccharides (EPSs), and mycosporine-like amino acids (MAAs). These molecules exhibit antioxidant, anti-inflammatory, photoprotective, and regenerative properties. This review analyzes the molecular adaptations that enable extremophiles to synthesize these compounds, and explores their cosmetic applications, including enzymatic exfoliation, UV protection, hydration, and anti-pollution effects. This paper examines their nutraceutical potential, highlighting systemic benefits such as improved skin elasticity, reduced photoaging, and modulation of the gut–skin axis via prebiotic EPSs. Industrial strategies for sustainable production, such as microbial fermentation, synthetic biology, and green extraction, are discussed. Examples of commercial ingredients like PlusXanthin™, Antarctic-G, and Desertica. Extremophile-derived ingredients combine biological efficacy with environmental sustainability, positioning them as key assets for next-generation skincare. Future directions include clinical validation, regulatory harmonization, and the development of personalized, microbiome-friendly formulations.

## 1. Introduction

In the pursuit of innovative and sustainable solutions for skin health, the cosmetic and nutraceutical industries are increasingly converging. This intersection has given rise to cosmeceuticals and nutricosmetics, formulations that combine topical and systemic approaches to promote skin resilience, beauty, and overall wellness. Within this evolving landscape, extremophiles capable of thriving in the most inhospitable environments on Earth are emerging as a novel and powerful source of bioactive compounds [1,2]. Extremophiles inhabit geochemically extreme niches such as deep-sea hydrothermal vents, hypersaline lakes, acidic springs, and polar ice [3]. Their ability to survive under conditions of intense heat, cold, pressure, dryness, salinity, radiation, and pH is made possible by unique molecular adaptations, including the production of extremozymes, extremolytes, and protective pigments [4]. These molecules not only ensure cellular integrity and DNA repair in harsh environments, but also exhibit properties highly desirable in skincare, such as antioxidant activity, UV protection, hydration, and anti-inflammatory effects [5,6]. The skin, constantly exposed to environmental stressors, undergoes structural and functional changes that accelerate aging, reduce elasticity, and compromise immune defense. Climate change and urban pollution further exacerbate these effects, making the need for adaptive and protective skincare more urgent than ever. Extremophile-derived compounds offer a biologically inspired solution: they work in harmony with the skin’s natural functions, enhance its ability to cope with stress, and possess excellent stability and compatibility with human metabolism [7]. Moreover, the evolutionary significance of extremophiles, some of which may represent the earliest forms of life, has led to renewed interest in their classification, with archaea proposed as a distinct domain due to their unique biochemistry and ribosomal RNA structure [8]. Their resilience and versatility have made them valuable not only in cosmetics, but also in medicine, biotechnology, and astrobiology [3,9,10,11]. From a biotechnological perspective, extremophile-derived compounds offer advantages in terms of sustainability, scalability, and compatibility with skin physiology. Their enzymes are already used in industrial processes such as PCR diagnostics, bioremediation, and drug synthesis [12]. In cosmetics, their long shelf life, metabolic compatibility, and ability to mimic natural skin defense mechanisms make them ideal candidates for next-generation formulations. This review provides a comprehensive overview of extremophile-derived bioactives with relevance to both topical and systemic skincare. We detail the molecular adaptations that underpin their biosynthesis; analyze their cosmetic and nutraceutical applications, including enzymatic exfoliation, UV protection, hydration, and gut–skin axis modulation; and discuss sustainable production strategies such as microbial fermentation and synthetic biology. By bridging cosmeceutical efficacy with environmental resilience, extremophiles emerge as a strategic resource for next-generation, science-driven skincare. In addition, this review advances beyond previous works by offering a comparative and multidisciplinary assessment linking molecular mechanisms, biotechnological production, and translational potential in cosmetic and nutraceutical fields.

## 2. Extremophiles as a Source of Bioactive Molecules

Due to their ability to survive in extreme environments, extremophiles have evolved molecular strategies that enable the production of highly stable and diverse bioactive compounds. These include enzymes, compatible solutes, and exopolysaccharides, which possess properties increasingly valued in health-related fields. Rather than revisiting their ecological diversity, this section focuses on the molecular adaptations that underpin their biosynthetic potential and sets the stage for exploring how these compounds can be harnessed in cosmeceutical and nutraceutical applications.

### 2.1. Adaptation Mechanisms to Extreme Temperatures and pH

**Figure 1:**
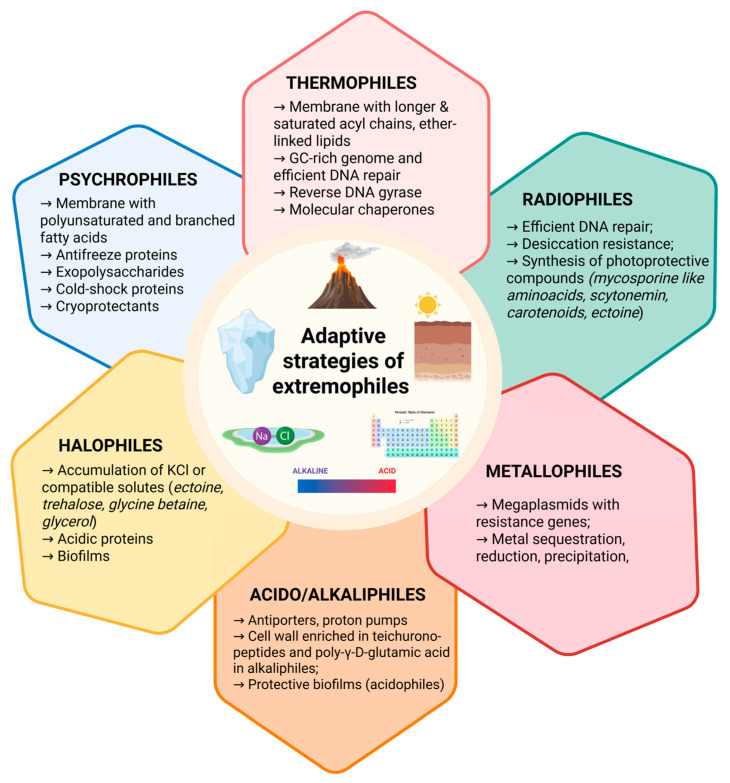
Overview of adaptive mechanisms in extremophiles. Extremophilic microorganisms have evolved diverse molecular, structural, and physiological strategies to thrive under extreme environmental conditions such as high temperature, salinity, pressure, acidity, and radiation.

Extremophiles exhibit a wide range of adaptive strategies that allow them to survive in environments considered hostile to most life forms, and these same mechanisms often underpin the production of bioactive compounds with unique properties relevant to cosmeceutical and nutraceutical applications (Figure 1 and Table 1).

#### 2.1.1. Cold Adapted

Organisms thriving in cold ecosystems, such as polar regions and deep-sea habitats, remodel their membrane lipids by incorporating polyunsaturated and branched fatty acids to preserve fluidity and nutrient transport at low temperatures. They also produce antifreeze proteins (AFPs) that inhibit ice nucleation and recrystallization, and EPSs that promote biofilm formation and locally alter freezing points [13,14]. Additional protection is provided by cold-shock proteins, which facilitate translation by destabilizing secondary RNA structures, and by cryoprotectants such as trehalose, glycerol, and betaine, which prevent protein aggregation and cellular damage [15]. At the genomic level, psychrophiles often display larger genomes with higher A + T content, enhancing replication efficiency under cold conditions [16]. Their enzymes exhibit high catalytic efficiency at low temperatures, achieved through increased molecular flexibility—characterized by reduced ionic interactions, fewer hydrophobic residues, enrichment in small side-chain amino acids (e.g., glycine, methionine), and elongated loops [17]. These adaptations result in bioactive molecules with direct relevance to cosmeceutical and nutraceutical applications. Trehalose and betaine, for example, are widely used in moisturizers and anti-aging products for their hydrating and protective properties [18,19]. Another notable compound is *ectoine*, a well-characterized extremolyte that reduces inflammation, protects against UV damage, and improves skin barrier function, making it suitable for sensitive skin and dermatological treatments [20]. EPSs from psychrophiles also contribute to skin hydration and anti-pollution effects by forming protective biofilms and modulating oxidative stress [21,22].

#### 2.1.2. Heat Adapted

At the opposite thermal extreme, thermophiles inhabit high-temperature niches such as hot springs, geothermal sites, and hydrothermal vents. To withstand thermal stress, they modify membrane lipids by increasing acyl chain length and saturation, stabilizing the bilayer in a liquid-crystalline state, and incorporating ether-linked lipids [23]. Genomically, they display compact genomes with reduced non-coding regions and elevated GC content, supported by efficient DNA repair systems. A key enzyme, reverse DNA gyrase, introduces positive supercoils that increase DNA melting temperature [16]. Their proteins are enriched in electrostatic interactions, salt bridges, disulfide bonds, and hydrophobic residues (e.g., valine, isoleucine, leucine, methionine), conferring thermostability and resistance to proteolysis [24]. Molecular chaperones assist in protein folding and repair under heat stress [25]. These features enable the production of extremozymes and chaperone proteins that remain active at elevated temperatures, and such enzymes are increasingly used in skincare formulations designed to protect against oxidative stress and UV damage.

#### 2.1.3. pH Adapted

Adaptations to extreme pH conditions reveal further biochemical ingenuity. Alkaliphiles, for example, maintain stable cytoplasmic pH levels in highly alkaline environments such as soda lakes and alkaline soils through Na^+^/H^+^ antiport systems, modifications to the secondary cell wall (e.g., teichurono-peptide and poly-γ-D-glutamic acid), and membranes enriched in branched-chain fatty acids and anionic phospholipids [26,27]. Intracellular acidification occurs via organic acid production and amino acid deamination, while membrane transporters and ATP synthases actively regulate ion balance. However, no bioactive molecules with cosmetic applications have been reported so far for this group. Conversely, acidophiles, which survive at pH 0–3, reduce proton permeability through membranes enriched in branched or cyclic fatty acids, including ether-linked archaeal lipids and pentacyclic triterpenoids [28,29]. Active proton efflux systems (ATPases, symporters, antiporters) and K^+^ uptake mechanisms help maintain near-neutral cytoplasmic pH [30]. Buffering is supported by basic amino acids and decarboxylase activity, while chaperones and DNA/protein repair systems mitigate acid-induced damage. Biofilm formation, regulated by quorum sensing, further enhances survival [31]. Acidophiles also combat elevated oxidative stress through the synthesis of organic antioxidants (e.g., glutathione) and enzymes like peroxidases, superoxide dismutases, and thioredoxins [32]. These antioxidant systems are of growing interest for cosmeceutical applications, particularly in formulations targeting oxidative damage and inflammation.

### 2.2. Adaptation Strategies to Salinity, Metals, Pressure, and Radiation

Extremophiles display a remarkable ability to adapt to a variety of extreme conditions, including high salinity, metal-rich environments, elevated hydrostatic pressure, and intense radiation. These adaptations not only ensure survival but also lead to the synthesis of bioactive molecules with significant potential for cosmeceutical and nutraceutical applications. In hypersaline environments, halophiles employ two main strategies: the accumulation of inorganic ions such as KCl and the synthesis of compatible solutes like glycine betaine, *ectoine*, trehalose, and glycerol [33,34,35]. These molecules stabilize proteins and membranes under multiple stressors, including salt, heat, desiccation, and cold. Halophilic proteins themselves are enriched in acidic surface residues, which enhance solubility and reduce aggregation, while biofilm formation further contributes to salt tolerance [36]. Many of these solutes, particularly *ectoine* and trehalose, have already found applications in skincare products for hydration, anti-aging, and protection against environmental stressors [37]. Organisms adapted to metal-rich environments, known as metallophiles, rely on megaplasmids encoding resistance genes and detoxify metals through mechanisms such as sequestration, precipitation, reduction, or volatilization [38,39]. These capabilities make them valuable for bioremediation and suggest potential nutraceutical applications aimed at heavy metal detoxification. Piezophiles, thriving under high hydrostatic pressure in deep-sea habitats, exhibit genomic and membrane remodeling, increased protein flexibility, and altered transcriptional regulation [40]. Although pressure-specific genes remain poorly defined, their enzymes demonstrate unique stability under mechanical stress, a feature that could be advantageous in cosmetic formulations requiring resilience during processing or application [41]. Finally, radiophiles and UV-resistant extremophiles, such as *Deinococcus radiodurans*, withstand ionizing and ultraviolet radiation through highly efficient DNA repair systems, protective proteins, and desiccation resistance mechanisms [42]. Marine UVR-extremophiles produce extremolytes and photoprotective metabolites such as mycosporine-like amino acids (MAAs), scytonemin, carotenoids, and *ectoine* [43,44]. These compounds are already incorporated into cosmeceutical products for UV protection, antioxidant defense, and skin barrier support, highlighting the direct relevance of these adaptations to skincare innovation [45,46,47].

## 3. Cosmetic Applications of Extremophile-Derived Compounds

**Figure 2:**
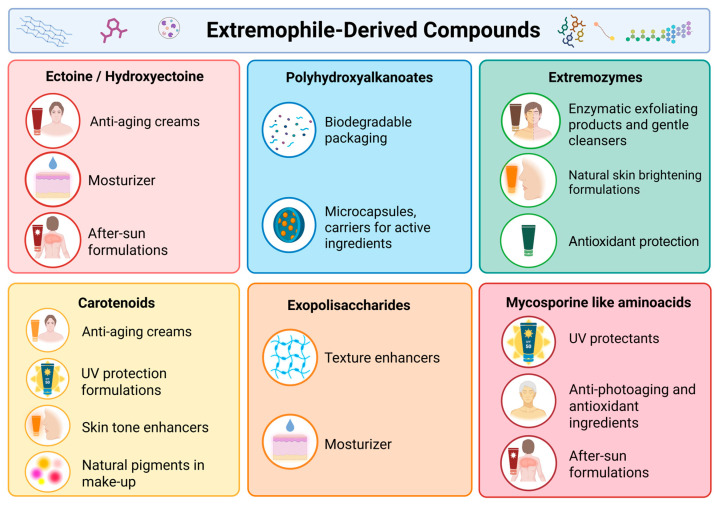
Cosmetic applications of extremophile-derived compounds including *ectoine*/*hydroxyectoine*, PHA, EPSs, extremozymes, carotenoids, MAAs, highlighting their roles in skin protection, UV.

Recent studies have shown an increasing interest in utilizing metabolites from extremophilic organisms in the cosmetics industry. These biomolecules, which have evolved under extreme environmental conditions, display unique functional properties, from chemical resistance to physical stress and excellent biocompatibility, that render them attractive as anti-aging, moisturizing, photoprotective, anti-pollution, and antioxidant actives. The following section will provide a detailed overview of the main molecular classes, including *ectoine*/*hydroxyectoine*, extremozymes, carotenoids, exopolysaccharides, PHA, and MAAs (Figure 2) [6,48,49].

### 3.1. Extremozymes

Extremophile-derived enzymes (i.e., extremozymes) are increasingly attractive in cosmetics because they remain active under harsh conditions and enable functions that are difficult for conventional enzymes [50]. Besides microbial enzymes, several formulations also employ enzyme complexes obtained from extremophile plants (e.g., xerophytes/halophytes) that are marketed as extremozymes. Experimental work indicates that these plant-derived complexes can mitigate UV- and pollution-induced damage, support DNA-repair–related responses, preserve proteostasis, and enhance skin resilience under environmental stress; proposed mechanisms include ROS quenching, chaperone-like stabilization of proteins and membranes, and the facilitation of DNA-repair pathways, such as reduced CPDs in UV-challenged models [15]. Beyond ROS quenching, topical DNA-repair enzymes (notably photolyase, derived initially from cyanobacteria/plants) have shown 40–45% reductions in CPDs in human skin when added to sunscreens, with multiple small but consistent clinical/irradiation studies supporting enhanced repair compared to sunscreen alone. These results align with the concept of extremozymes, which involves stress-adapted enzymatic protection [51,52]. When compared to conventional enzymes used in cosmetic formulations or nutraceutical processing (e.g., lipases, proteases from mesophilic sources), extremozymes exhibit enhanced catalytic efficiency and remarkable stability across extreme temperature, salinity, and pH ranges [8,9]. These properties enable more robust and sustainable manufacturing processes, reducing energy consumption and the need for stabilizing additives. Although their superior performance under industrial conditions may compensate for initial production costs in high-value formulations, large-scale application is still affected by the high costs of production and purification, as well as limited data on their immunogenicity and safety in humans.

#### 3.1.1. Proteases

Proteases (e.g., thermo-alkaline subtilisins from thermophilic bacteria) act as enzymatic exfoliants. By hydrolyzing adhesive proteins in corneodesmosomes, they promote gentle desquamation and support physiological epidermal turnover, resulting in lower irritation compared to typical chemical or mechanical peels [12,53,54]. Extremozymes also retain activity across expansive pH/temperature windows and tolerate solvents, salts, and preservatives—properties documented for proteases from hyperthermophiles and halo-alkaliphiles [50,55]. Clinically oriented reviews of enzymatic peels report measurable improvements in surface smoothness and low-irritation profiles versus classic (AHAs), consistent with protease action on corneodesmosomes [54]. From a stability standpoint, bromelain (a model protease) retained measurable proteolytic activity for 180 days when incorporated in emulsions (more than in gels) under controlled storage, underscoring the importance of matrix and excipients in preserving enzyme function [56]. Alkaline proteases from *Bacillus* spp. routinely reach ~350–2000 U/mL in submerged fermentations after optimization (with casein/carbon–nitrogen balance), providing a scalable supply for topical uses [57].

#### 3.1.2. Lipases

Lipases (e.g., lipase B from *Candida antarctica*, isolated from psychrophilic environments) show solvent stability useful for synthesizing emollient esters and fragrances. They can serve as sebum-modulating cofactors in oily-skin products by controllably hydrolyzing triglycerides in sebum [58,59]. In oily-skin concepts, exogenous lipases are being explored to modulate surface lipids; in parallel, inhibiting microbial lipase activity (e.g., from *Cutibacterium acnes*) is a recognized strategy to limit pro-inflammatory free fatty acids, highlighting the need for dose-controlled lipase use on the skin [60,61]. Manufacturing robustness is high: *Candida antarctica* lipase B is a workhorse biocatalyst, valued for solvent tolerance and thermal stability in non-aqueous systems (used broadly to make cosmetic esters), which translates to good formulation resilience [62,63].

#### 3.1.3. Amylases

Amylases from thermo-/psychrophilic bacteria contribute to deep cleansing/anti-pollution concepts by degrading residual polysaccharides (e.g., starch traces, biofilm exopolymers) on the skin surface, improving removal of particulate matter and impurities [64,65]. Thermo/alkali-stable *Bacillus* amylases typically deliver ~130–250 U/mL in optimized submerged fermentations (35–48 °C; pH 5.5–8), and retain functional activity around 60 °C, making them compatible with cleanser manufacturing and storage [66,67,68].

#### 3.1.4. Chitinases

Chitinases (e.g., from alkaliphilic actinomycetes) are being explored in anti-dandruff shampoos and lotions because they hydrolyze fungal chitin (e.g., in *Malassezia*), thereby reducing colonization and associated flaking/itch [69,70,71]. While published cosmetic clinical data are still limited, the antifungal rationale is strong given that *Malassezia*-associated scalp disease is typically managed by targeting the fungus and inflammation. Moreover, chitooligosaccharides, the bioactive fragments generated by chitinase, show anti-inflammatory and antioxidant activity (reduction in NF-κB/IL-1β/IL-6/TNF-α levels), supporting scalp-soothing benefits alongside antifungal action. [72,73].

Overall, extremozymes offer high thermal/chemical stability, functionality at extreme pH, biodegradability, and sustainable production (fermentation, including engineered extremophiles on waste carbon). Limitations include possible irritation/allergenicity (mitigated by purification and post-use deactivation), upstream costs for some enzymes, cultivation hurdles for specific extremophiles, and regulatory safety testing for proteins [50]. Despite these caveats, the use of extremophile enzymes in “biotech” cosmetics is growing, with protease-based enzymatic peels already in use and increasing interest in lipases and other classes [12].

### 3.2. Ectoine

*Ectoine* ((4S)-2-methyl-1,4,5,6-tetrahydropyrimidine-4-carboxylic acid) is a small molecule identified in a wide range of halophilic and halotolerant bacteria due to their ability to balance the intra- and extracellular osmotic pressure (Paragraph 2.2) [74]. This compatible solute is a cyclic derivative of aspartate and commonly it is produced in mixture with the *hydroxyectoine* (1,4,5,6-tetrahydro-2-methyl-5-hydroxy-4-pyrimidinecarboxylic acid), which is the hydroxylated derivative of *ectoine* [75]. The biological role of *hydroxyectoine* explains why it is produced alongside *ectoine*, as it serves as a more effective desiccation protectant [76]. Generally, these compounds are not only osmotic protective agents but also biofunctional stabilizers and skin-protective agents for the treatment of airway inflammatory conditions such seasonal allergic rhinitis symptoms, including *ectoine* in nasal spray and eye drops [77,78]. Moreover, *ectoin* represents a safeguard against oxidative stress induced by H_2_O_2_ and UV-A radiation, as evidenced in a model system involving human skin fibroblasts, upregulating the PI3K/AKT signaling pathway [79]. The biosynthesis of *ectoine* begins with L-aspartate. It is carried out by five sequential enzymatic reactions, carried out by l-aspartate kinase (Ask), l-aspartate-β-semialdehyde dehydrogenase (Asd), l-2,4-diaminobutyrate aminotransferase (EctB), l-2,4-diaminobutyrate acetyltransferase (EctA) and synthase (EctC) [80]. The first two enzymes (Ask and Asd) are the canonical for the biosynthesis of aspartate whereas EctB, EctA and EctC are present in a gene cluster (ectABC) and the expression is activated by high concentration of salts [75]. The microbial production of the *ectoine*/*hydroxyectoine* by several halophiles belonging mainly to the family of Halomonadaceae, reaching (0.15–0.2 g/g dry cells) such as *H. salina* DSM5928 (0.14 g/g _glucose_), *H. elongata* DSM142 (0.11 g/g _monosodium glutamate_), *H. hydrothermalis* Y2 (0.21 g/g _glucose and monosodium glutamate_) [37,81]. However, from an industrial point of view, the ectoin production is influenced by three diverse factors: (i) the current bioproduction process for yields a mixture of *ectoine* and *hydroxyectoine*, which are chemically similar compounds and challenging to separate; (ii) the high concentrations of NaCl required to induce the production in such halophiles negatively impact the performance and durability of the equipment used; (iii) the strain currently used for ectoine production exhibits a relatively low yield [37]. For the first point, Orhan et al. established a novel sustainable protocol using acid and base in the purification process of *ectoine*, growing a novel strain of *Piscibacillus halophilus* as an alternative *ectoine* producer [76] obtaining *ectoine* with high purity (99.15%) and yield (99%). Therefore, synthetic biology approaches that use heterologous hosts such as *Escherichia coli, Corynebacterium glutamicum*, *Hansenula polymorpha* and *Pichia pastoris* represent a promising alternative. These systems allow for the targeted expression of specific genes, also selecting genes from metagenome data from extreme environments, enabling high-yield production of pure *ectoine* [82,83]. Such synthetic biology-based systems not only provide a sustainable route to produce ectoine at high purity and yield, but also open the door to innovative applications. For instance, when used in combination, ectoine enhances the moisturizing and anti-inflammatory effects of hyaluronic acid, resulting in longer-lasting hydration, improved skin barrier recovery, and greater protection against irritation compared to hyaluronic acid alone [84]. This synergistic formulation offers a biomimetic and sustainable alternative to conventional synthetic moisturizers and stabilizers, combining cellular protection with environmentally friendly production through microbial fermentation.

### 3.3. Carotenoids

Most of the carotenoids currently available on the market are obtained through vegetable extraction or chemical synthesis. However, these production routes face several limitations, including seasonal and geographical variability, low yields, and environmental concerns associated with large-scale cultivation and solvent use [85]. Extremophilic microorganisms from all three domains (Archaea, Bacteria, and Microalgae) produce distinctive carotenoids in response to environmental stress, such as high salinity, temperature, or radiation. These adaptive mechanisms make extremophiles valuable microbial cell factories for the sustainable and controlled production of carotenoids with enhanced stability and bioactivity. The radioresistant bacterium *Deinococcus radiodurans*, for example, produces the unique xanthophyll deinoxanthin [86]. While it has been demonstrated that halophilic archaea (e.g., *Haloferax*, *Halobacterium* spp.) have the capacity to accumulate the rare carotenoid bacterioruberin. It has been shown that bacteria that have adapted to low temperatures and ultraviolet (UV) radiation, for example, Arthrobacter sp., are capable of synthesising pigments such as decaprenoxanthin and sarcinxanthin [87]. Extremophilic microalgae have also been shown to be prolific carotenoid producers. For example, the halotolerant green alga *Dunaliella salina* has been observed to store up to ~10% of its dry weight as β-carotene under conditions of high light and high-salinity stress [66]. In comparison, the freshwater alga *Haematococcus pluvialis* has been found to accumulate up to ~4% dry weight as astaxanthin in its red cysts [88]. These pigments fulfil well-established adaptive functions, namely as potent antioxidants and UV-absorbers. They act to protect cellular components from photodamage and oxidative stress, and they intercalate into membranes to maintain fluidity under extreme conditions. For instance, DNX is a potent quencher of singlet oxygen and hydrogen peroxide [89]. Bacterioruberin’s extended conjugation and polar hydroxyl groups give it unusually high antioxidant capacity and the ability to stabilize halophilic membranes against osmotic stress. In practice, extremophiles characterized by a high pigment content exhibit a capacity to withstand otherwise lethal insults, such as UV or oxidative stress. For instance, *Arthrobacter* strains isolated from Antarctic soils utilize carotenoids to enhance UV-B resistance [87], and human skin cells treated with DNX show markedly reduced UV-B-induced apoptosis and improved collagen synthesis [86]. The multifaceted protective characteristics of extremophile carotenoids render them a compelling prospect for incorporation into skincare formulations. Extremophile-derived carotenoids have been shown to offer antioxidant, anti-ageing, photoprotective, and “anti-pollution” benefits. The quenching of ROS has been demonstrated to limit photoaging and preserve barrier lipids. Furthermore, their intrinsic UV absorption has been shown to provide a natural form of protection [86,87]. DNX (PlusXanthin™) has been demonstrated to reduce wrinkle formation and inflammation in skin models. At the same time, β-carotene and astaxanthin are frequently employed as key ingredients in anti-aging formulations due to their strong free-radical scavenging properties (*H. pluvialis* astaxanthin is often referred to as a “potent antioxidant” [90]). Bacterioruberin-rich extracts have been shown to provide UV shielding and counteract glycation, with reports of increased collagen and ceramide levels in treated cells. To summarise, carotenoids originating from extremophiles constitute a distinctive class of bioactive substances. The structural diversity of these substances, coupled with their evolutionary optimization in harsh conditions, results in remarkable stability and multifunctionality in skin care applications. The evidence suggests that these substances have antioxidant, photoprotective, and anti-ageing effects. Additionally, a growing body of research supports the use of scalable production strategies derived from microalgae, bacteria, and archaea. This positions them as sustainable and high-value ingredients for next-generation cosmeceuticals.

### 3.4. Mycosporine-like Amino Acids

Mycosporine-like amino acids (MAAs) are a class of secondary metabolites that are widely produced by extremophilic microorganisms, particularly marine algae (especially red seaweeds), cyanobacteria and certain fungi [91,92].

Commercial formulations containing mycosporine-like amino acids are already available on the market, such as Helioguard™ 365 (Mibelle Biochemistry, Switzerland), derived from Porphyra umbilicalis and designed for UVA protection and anti-aging applications, and Helionori^®^ (Gelyma, France), a marine extract enriched in MAAs that provides natural photoprotection and skin defense against oxidative stress [93]. These examples highlight the growing interest in MAAs as natural UV-screening compounds and suggest that extremophilic marine microorganisms represent a reservoir to produce novel potential MAAs. Chemically, they are small (<400 Da), water-soluble, and colourless molecules characterized by a cyclohexenone or cyclohexenamine ring that is conjugated to an amino acid or imino alcohol substituent [91]. Over 30 distinct MAAs have been identified, all sharing this core structure that is responsible for intense ultraviolet (UV) absorption [91]. These compounds typically absorb maximally in the UV-A/UV-B range (309–362 nm) with high molar extinction coefficients (ε ≈ 28,000–50,000 M^−1^ cm^−1^ ) effectively acting as natural ‘microbial sunscreens’ to shield organisms from solar radiation. In their native hosts, MAA levels rise in response to intense UV exposure or other stresses, reflecting an adaptive photoprotective function in extreme environments [92]. MAA biosynthesis is linked to primary metabolic pathways and involves unique enzyme clusters. Early evidence in coral symbionts suggested that the shikimic acid pathway was involved in forming the cyclohexenone core (for example, glyphosate inhibits shikimate metabolism, which blocks MAA accumulation). More recently, however, a pentose phosphate pathway (PPP) origin has been demonstrated. A PPP-derived intermediate (sedoheptulose-7-phosphate) is converted into the key precursor 4-deoxygadusol via a series of enzymatic steps. This is then cyclised and conjugated with amino acids to yield various MAAs [94]. In cyanobacteria, a conserved six-gene cluster encodes enzymes for MAA assembly, including a sugar cyclase (e.g., 2-epi-5-epi-valiolone synthase), an O-methyltransferase, an ATP-Grasp ligase and a non-ribosomal peptide synthetase (NRPS)-like enzyme, which sequentially constructs the MAA scaffold and attaches specific amino acid residues [95]. Notably, the heterologous expression of a four-gene Abiosynthetic cluster from the cyanobacterium Anabaena variabilis in *E. coli* was sufficient to produce shinorine, a MAA, demonstrating the feasibility of fermentative MAA production via metabolic engineering [41]. In parallel, biotechnological cultivation strategies are employed to increase MAA yields in native producers. For example, macroalgae can be farmed in nutrient-rich, high-UV conditions (e.g., in an integrated multi-trophic aquaculture system with supplemental UV exposure) to stimulate MAA biosynthesis for sustainable commercial harvesting. Such approaches address the limited natural abundance of MAAs, enabling a scalable supply of these UV-absorbing compounds for cosmeceutical use [96]. Their accumulation has been observed in response not only to UV stress but also to salt and desiccation stress. The saline and hypersaline environments led to the accumulation of MAAs in several cyanobacteria [97,98]. Already 30 years ago, several cyanobacteria were discovered to accumulate MAAs, such as the unicellular cyanobacteria ‘Euhalothece’ type producing MAA-2-glycine, with maximum absorbance at 331 nm and a novel compound with maximum absorbance at 362 nm (‘euhalothece-362’), recently identified as 2-(E)-3-(E)-2,3-dihydroxyprop-1-enylimino-mycosporine-alanine [98]. Another cyanobacteria, *Chlorogloeopsis* strain PCC 6912, tolerates salt up to 70% seawater salinity, and produces shinorine and MAA-glycine. Moreover, halotolerant fungi, including *Cladosporium sphaerospermum*, *Cladosporium cladosporioides*, the halophilic black yeasts *Phaeotheca triangularis* and *Hortaea werneckii*, as well as the halotolerant *Aureobasidium pullulans*, produce high content of MAA-glutamicol-glucoside, whereas black yeasts also produce smaller amounts of MAA-glutaminol-glucoside. In line with this, colonies of black melanized fungi (*Sarcinomyces*, *Coniosporium*, *Phaeotheca*) inhabiting desert rock surfaces, where they are exposed to desiccation, intense UV radiation, and nutrient scarcity, were found to contain high concentrations of MAA-glutaminol-glucoside [98]. In the domain of skincare applications, MAAs function as broad-spectrum photoprotective and antioxidant agents at the molecular level. The conjugated ring systems of these molecules absorb harmful UV-A and UV-B photons, thereby dissipating the energy as heat. This process prevents direct DNA damage (e.g., cyclobutane pyrimidine dimer formation) without generating reactive photoproducts [95,99]. These compounds have also been shown to counteract oxidative stress actively. MAAs have been shown to scavenge UV-induced reactive oxygen species and enhance cellular antioxidant defenses (e.g., by increasing the activity of enzymes such as superoxide dismutase and catalase) [95]. It is through such mechanisms that MAAs mitigate the cascade of UV-induced cell injury, apoptosis, and inflammation. For instance, topical MAAs (e.g., shinorine and porphyra-334 from red algae) enhanced skin antioxidant enzyme levels and inhibited UV-triggered inflammatory signalling, thereby protecting keratinocytes from UV-induced apoptosis. It has been demonstrated that certain MAAs specifically absorb the shorter UV wavelengths that drive pro-inflammatory cyclooxygenase-2 (COX-2) upregulation [92,95]. This process effectively suppresses UV-induced COX-2 expression and subsequent inflammation. Furthermore, MAAs contribute to the preservation of the skin’s structural integrity by preventing photoaging at the level of the extracellular matrix. Research conducted on UV-exposed skin models has demonstrated that MAAs have the capacity to inhibit matrix metalloproteinases (MMPs), including collagenase MMP-1 and elastase, which are known to be elevated by UV stress. This effect of MAAs is characterised by a reduction in collagen degradation and a concurrent promotion of new collagen synthesis in the dermis. This multifaceted mode of action, combining UV filtration, antioxidant defense, anti-inflammatory effects, and matrix protection, underlies the value of MAAs as holistic skin protectants. MAAs have garnered attention due to their protective properties, rendering them a promising natural ingredient in cosmeceuticals focused on photoprotection and anti-aging. They are regarded as safe, biodegradable alternatives to synthetic sunscreen chemicals, exhibiting low toxicity and exceptional photo- and thermostability (no toxic breakdown products even after prolonged UV exposure). Several MAA-rich extracts from extremophile organisms have already been commercialized in the skincare industry. Indeed, the incorporation of MAAs into next-generation sunscreens and skincare products (e.g., “reef-safe” sunscreens and anti-photoaging creams) is an emerging trend that seeks to bridge sustainable nutraceutical-inspired biocompounds with advanced skincare science for holistic skin wellness [92].

### 3.5. Exopolysaccharides and Polyhydroxyalkanoates

Microbial EPSs are produced by polyextremophiles in adaptation to multiple extremes (desiccation, temperature and high-pressure stresses). Each microorganism produces a unique EPS, and this extreme diversity translates into biologically infinite possibilities [100]. In comparison with conventional polysaccharides, including hyaluronic acid or xanthan gum, extremophilic EPSs can often demonstrate superior rheological properties, thermal stability, and additional bioactivities (antioxidant, anti-inflammatory). Moreover, bacteria can produce EPSs in large quantities starting from industrial wastes through biotechnological processes with a high production yield. Several marine and extremophilic bacteria have been reported to produce EPSs with distinctive structural and functional features. Benhadda et al. provided a comprehensive overview of marine bacteria-derived exopolysaccharides (EPSs), highlighting several that have already been commercialized in cosmetic formulations, including Epidermist 4.0™, EPS Bright™, EPS Seafill™, EPS Seamat™, EPS Seaglow™, and EPS Seapur™ (Codif Technologie Naturelle, Saint-Malo, France); Hyadisine^®^ and Hyanify^®^ (Lubrizol, Wickliffe, OH, USA); as well as Abyssine™ PF, Exo-H™, Exo-P™, and Exo-T™ (Lucas Meyer, Québec, QC, Canada) [101].

Recently, novel microorganisms isolated from extreme environments have shown the peculiar ability to produce EPSs. A recent example is the extremophile *Vreelandella titanicae* Zn11_249 [102] from Salar de Uyuni, which produced distinct EPSs under kosmotropic and chaotropic salt stress (Table 2).

These polymers differed in composition and molecular weight, exhibited strong antioxidant activity, lacked cytotoxicity, and showed promising potential for biomedical and cosmetic applications. Moreover, several thermophilic isolates (*Geobacillus, Parageobacillus, Aeribacillus,* and *Anoxybacillus*) yielded EPSs [103] with antioxidant, antibacterial, prebiotic, and antibiofilm properties, further underscoring their biotechnological relevance in health and food sectors. An immunoactive EPS was extracted from the Antarctic *Pseudoalteromonas* sp. LP6-12-2 [104] his branched heteropolysaccharide, mainly composed of mannose, significantly enhanced macrophage proliferation, phagocytosis, enzyme activity, and cytokine production, highlighting its potential as a novel immunomodulator for functional food and pharmaceutical applications. Similarly, *Halomonas* sp. DT-Z4 [105], a moderately halophilic strain isolated from saline and alkaline soil, produced a fructose-dominated heteropolysaccharide (EPS-Z4) with high thermal stability, excellent water solubility, and strong oil retention capacity. *Alkalihalobacillus* sp. [106], a multi-stress tolerant bacterium from bauxite residue, produced EPS under alkaline conditions, while *Bacillus* sp. NIOTSM16 [107] from seamounts synthesized EPS containing monosaccharides (glucopyranoside, ribofuranose, fructose) and fatty acids (octanoic, hexadecanoic, octadecanoic acids). Not only bacteria, but also fungi and microalgae produce EPSs. Recent examples include EPS-BMD26 from the cold-adapted yeast *Rhodotorula glutinis*, [108] a β-D-glucan (118 kDa) with high thermal stability, porous semicrystalline structure, good solubility, antioxidant activity, and strong antibiofilm effect against *S. aureus*, showing potential as a biohydrocolloid, antibiofilm agent, and food additive. EPSs from the psychrotolerant microalga *Chlamydomonas* sp. ASYA25 [109] were characterized and tested as cryoprotectants. At 5%, these EPSs enabled optimal recovery with low toxicity and preserved membrane integrity, suggesting microalgal EPSs as sustainable alternatives to conventional cryoprotectants. EPSs produced by extremophilic bacteria, microalgae and fungi, which are adapted to survive in environments with high levels of salinity, radiation, cold or pressure, have unusual sugar compositions (e.g., uronic acids and sulfation), a high anionic charge density and branched architectures [110]. These properties give them strong water-binding, film-forming and barrier capabilities that are relevant to skincare. In cosmetics, both bacterial and marine EPSs act as active ingredients (e.g., moisturising, soothing, anti-pollution and antioxidant) and as functional ingredients (e.g., rheology modifiers and texture enhancers) [101]. Recent reviews document the rapid growth of EPS-based skincare actives and clarify how structural motifs (e.g., charge, branching, sulphate groups) influence water retention, substantivity and the binding of particulate pollutants and metal ions to the skin’s surface [101,111]. Mechanistically, EPSs increase the hydration of the stratum corneum by forming hydrogen-bond networks and semi-occlusive films that reduce transepidermal water loss (TEWL). Several EPSs also increase the expression of markers of epidermal hydration and barrier function. For example, one EPS derived from marine microbes increased AQP3 and filaggrin (FLG) mRNA by a factor of between 1.6 and 3.2 in keratinocyte models, thereby supporting improved water transport and barrier maturation [112]. Extremophilic exopolysaccharides (EPSs) can provide additional protection against oxidative and radiation stress. For example, the EPS produced by *Deinococcus radiodurans* (a bacterium that is resistant to UV and gamma radiation) has been shown to shield human skin cells from irradiation-induced damage and reduce ROS-mediated injury. This is consistent with the intrinsic radioprotective ecology of the producer [113]. Collectively, these activities align with ‘anti-pollution’ claims, such as the adsorption/chelation of particles, and the mitigation of pollution-related oxidative stress, as well as with moisturization claims, including the reduction in TEWL and the support of AQP3/FLG. Furthermore, marine/halophilic sourcing is aligned with the drivers of the blue bioeconomy and sustainability [114]. From a manufacturing perspective, the production of extremophilic EPSs requires control of salinity, temperature and shear during fermentation. Nevertheless, recent overviews have highlighted scalable processes and downstream options (including more environmentally friendly extraction methods) that are tailored to the highly viscous broths that are typical of EPS production [49,115].

Polyhydroxyalkanoates (PHAs), which include biopolymers such as polyhydroxybutyrate (PHB) and polyhydroxyvalerate (PHBV), are valued for their biocompatibility, biodegradability and low toxicity. They are increasingly being considered for use in cosmetics, for example, as skin-contact films, mask substrates, microbead replacements and delivery matrices [116,117].

The well-known halophilic bacteria *Halomonas* spp. and *Paracoccus* spp. are the most important and extensively studied for their ability to accumulate high PHA yields under saline and stress conditions [118]. These microorganisms possess robust metabolic pathways that enable efficient polymer synthesis from diverse and low-cost substrates. From a biotechnological perspective, several low-cost carbon sources have been utilized for PHA production, including industrial and biodiesel byproducts (e.g., crude glycerol, waste frying oil, and algal biodiesel residues), agro-industrial and lignocellulosic residues (e.g., wheat straw, bamboo powder, and bagasse extract), and food processing or fermentation effluents (such as cheese whey, leguminous and fruit processing waters, and other agro-industrial effluents) [119]. These alternative feedstocks not only reduce production costs but also contribute to waste valorization and circular bioeconomy strategies, highlighting the sustainable potential of extremophilic bacteria in biopolymer production.

PHA films, in either leave-on or rinse-off formats, provide breathable, flexible coverage and a pleasant skin feel, as well as controlled occlusion. PHA/starch blends have been demonstrated to be effective as beauty mask supports, offering good skin compatibility and mechanical performance [120]. In terms of the environment, PHA microbeads demonstrate biodegradation in seawater and deep-sea environments, and have comparable mechanical performance to petroleum beads. This positions PHAs as a credible alternative to microplastics in exfoliants and texturising beads [121]. The pressure from regulators and consumers to phase out persistent microplastics is further accelerating interest in PHA-based systems for personal care products. As well as their structuring function, PHAs can be used to deliver cosmetic actives in the form of microparticles, nanofibres or films, supporting sustained release and dermal compatibility. PHBV/PHB nanofibre or composite dressings are cytocompatible and pro-regenerative in skin models, demonstrating their safety at the skin interface [122,123,124,125]. Although cost and brittleness remain challenges, the development of new materials and copolymer blends (e.g., PHBV plasticised films) is expanding the range of materials available for use in cosmetics [126,127]. Overall, extremophilic EPSs offer moisturisation, anti-pollution and mitigation of oxidative stress via film formation and support of barrier genes, while PHAs provide sustainable, skin-compatible structuring and delivery platforms (as well as microbead alternatives), which are aligned with clean beauty and circularity goals. Together, they enable robust, eco-designed formulations for next-generation skincare products [101,128]. From a regulatory perspective, several extremophile-derived compounds have an established safety record. For example, ectoine is listed in the EU CosIng database for cosmetic use, and carotenoids such as β-carotene and astaxanthin are approved by EFSA as food additives under Regulation (EC) No 1333/2008. These classifications confirm their suitability for topical and oral applications, supporting their integration into cosmeceutical and nutraceutical products. Overall, the results discussed in the previous sections highlight the remarkable potential of extremophile-derived compounds as multifunctional ingredients for cosmeceutical and nutraceutical applications. Their unique structural and physicochemical features enable a wide range of biological activities, that often exceed those of conventional synthetic counterparts. Despite the current technological and regulatory limitations, the growing interest in sustainable and biologically inspired solutions strongly supports further exploration and industrial translation of these natural bioactives. Therefore, despite the limitations summarized in Table 3, continued advances in bioprocess engineering, synthetic biology, and safety assessment will be crucial to fully exploit the potential of extremozymes while ensuring both environmental and consumer safety.

## 4. Nutraceutical Potential of Extremophile-Derived Compounds

**Figure 3:**
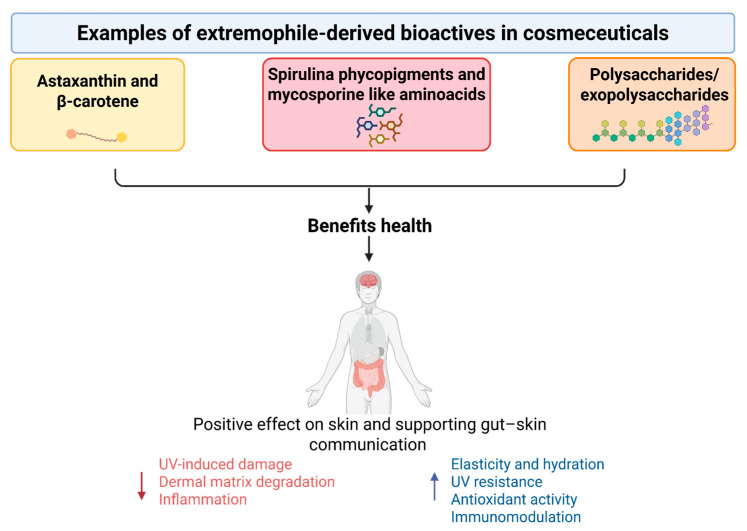
Extremophile-derived nutraceuticals enhance skin health by increasing elasticity, hydration, and UV protection, while reducing oxidative stress and inflammation.

Nutraceuticals derived from extremophiles, most notably marine/cyanobacterial carotenoids (e.g., astaxanthin from *Haematococcus pluvialis*, β-carotene from *Dunaliella salina*), cyanobacterial metabolites (e.g., Spirulina phycopigments/MAAs), and marine polysaccharides/exopolysaccharides, have been shown to have systemic antioxidant, anti-inflammatory, and immunomodulatory effects that can lead to measurable benefits for the skin and overall wellness (Figure 3).

Clinical studies and meta-analyses suggest that taking 4–12 mg of oral astaxanthin per day for 6–8 weeks improves multiple photoaging indicators, including skin elasticity, moisture, and transepidermal water loss, and reduces UV-induced damage [129]. These effects are further enhanced when oral astaxanthin is combined with topical application, supporting a systemic–topical synergy model [130,131]. Other clinical photoprotection trials also demonstrate that carotenoid supplementation increases the minimal erythema dose and reduces UV-induced erythema. This is consistent with the quenching of systemic antioxidants and the stabilisation of cell membranes [132,133]. Controlled field studies with *D. salina* extracts under high solar exposure report decreased skin glycation and improvements in photoaging biomarkers, which exceed those of *H. pluvialis*. This aligns nutraceutical intake with anti-glycation/antioxidant mechanisms relevant to dermal matrix preservation [134]. At a mechanistic level, extremophilic carotenoids and MAAs scavenge reactive oxygen species, modulate redox-sensitive transcription (e.g., NF-κB) and reduce inflammatory mediators (e.g., COX-2, IL-1β/IL-6). This dampens apoptosis and the activity of matrix metalloproteinases, which are implicated in collagen degradation. While most evidence for MAAs is preclinical, topical and semisystemic studies using Porphyra-derived MAAs in UV-challenged models demonstrate increased levels of endogenous antioxidant enzymes (SOD and CAT) and reduced inflammatory signalling. This supports their potential for use as oral–topical photoprotectants, pending clinical validation [95,99]. Bacterioruberin, an archaeal carotenoid endemic to halophiles, exhibits potent antioxidant and immunomodulatory activities in vitro and in vivo. Work is advancing on formulations to stabilise its labile, highly conjugated structure, positioning it as a next-generation nutraceutical pigment for systemic photoprotection and inflammation control [5,135]. Cyanobacterial biomass from Arthrospira/Spirulina offers a pragmatic, extremophile-based nutraceutical. There is human data to suggest that it has immune-modulating and anti-inflammatory effects, which are attributed to phycocyanin, carotenoids and polysaccharides. Evidence from randomised and synthesised studies indicates improvements across inflammatory and allergic endpoints. However, condition-specific responses vary, and there are isolated reports that caution against immune-stimulating contexts for all patients. This underscores the need for indication-tailored use [136,137,138]. Marine and extremophilic exopolysaccharides (EPSs) function as prebiotic-like modulators of the gut microbiota, enhancing short-chain fatty acid production, strengthening epithelial barriers and reducing endotoxin-driven inflammation. These mechanisms are closely linked to the ‘gut–skin axis’ via cytokine and metabolite signalling, which influences atopic dermatitis, psoriasis flare-ups and photoaging phenotypes [139,140]. Recent clinical and translational overviews of the gut–skin axis confirm that probiotics and prebiotic polysaccharides can enhance skin health by addressing dysbiosis and reducing systemic inflammation, thereby establishing a link between the consumption of extremophilic EPSs and dermatological outcomes [141]. Taken together, the literature supports a dual-route strategy in which extremophile-derived nutraceuticals provide antioxidant and anti-inflammatory coverage from the inside out, complementing topical actives from the outside in. Meta-analytic and trial data on astaxanthin explicitly document the additional benefits of combining oral and topical regimens for improving skin elasticity, moisture and UV resilience. This provides a template for future clinical designs involving MAAs, bacterioruberin and EPS-based prebiotics [131]. Priority gaps include head-to-head dosing studies, standardized biomarkers (e.g., MED, collagen/elastin turnover, TEWL), and longer-term safety/efficacy trials for newer extremophile metabolites. Nonetheless, the convergence of systemic antioxidant capacity, immune calibration via the gut–skin axis, and synergy with topical delivery positions extremophile nutraceuticals as credible candidates for holistic, evidence-based skin health strategies [131,139,140].

## 5. Cosmeceutical Integration and Sustainability

**Figure 4:**
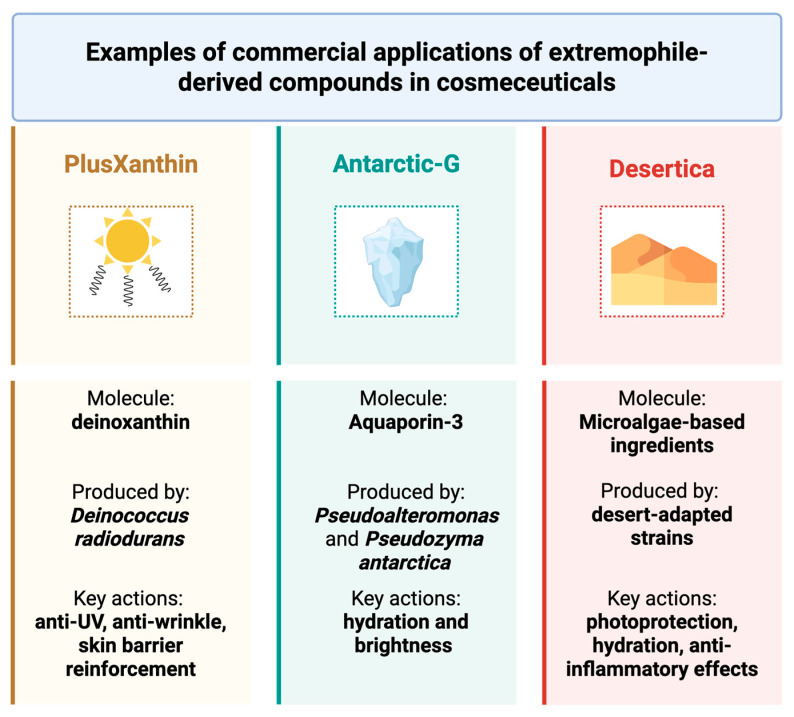
Examples of commercial applications of extremophile-derived compounds in cosmeceuticals: PlusXanthin from *Deinococcus radiodurans*, Antartic-G from *Pseudoalteromonas* and *Pseudozyma antarctica*, Desertica from desert-adapted strains.

The integration of extremophile-derived bioactives into cosmeceutical formulations represents a paradigm shift toward sustainable innovation in skincare. These bioactives, sourced from organisms thriving in extreme environments, offer natural resilience, stability, and multifunctionality, reducing the reliance on synthetic additives and preservatives. Their biotechnological production often relies on renewable resources and low-impact processes, such as microbial fermentation and controlled biomass cultivation, which minimize energy and water consumption. A notable example is PlusXanthin, derived from *Deinococcus radiodurans*, whose carotenoid deinoxanthin is encapsulated to enhance antioxidant efficacy and stability (Figure 4).

Clinical studies report a reduction in under-eye fat (−11.6%), improved skin density (+7.5%), and lightening of dark circles (+13.4%) after four weeks [33]. Its multifunctionality—anti-UV, anti-wrinkle, and skin barrier reinforcement—makes it ideal for sensitive areas, while its microbial origin supports eco-friendly sourcing. Similarly, Antarctic-G, obtained from *Pseudoalteromonas* and *Pseudozyma antarctica*, contains glyceryl glucoside (GG) and EPSs that activate Aquaporin-3, enhancing hydration (+37.4%) and brightness (+4.2%) as reported by manufacturers [142]. These postbiotic compounds are produced via fermentation-based bioprocesses, which are scalable and environmentally benign. Microalgae-based ingredients, such as *Desertica*, cultivated from desert-adapted strains, offer photoprotection, hydration, and anti-inflammatory effects [143]. Their cultivation requires minimal inputs and can be integrated into carbon-neutral systems, contributing to CO_2_ mitigation and land-use efficiency.

**Figure 5:**
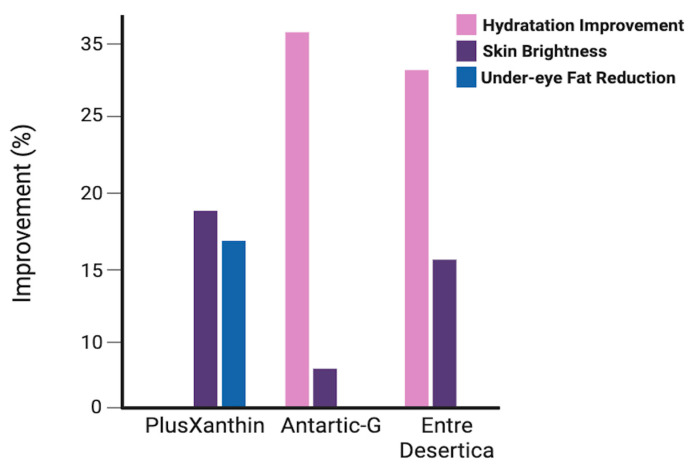
Efficacy metrics of extremophile-derived ingredients.

Among the extremophile-derived ingredients analyzed, as shown in Figure 5, Antarctic-G stands out for its superior hydrating properties, making it particularly suitable for formulations aimed at protecting the skin from environmental stress. Desertica, derived from desert-adapted microalgae, is especially effective in enhancing skin brightness and combating dryness, positioning it as an ideal candidate for sun-exposed or arid skin types. In contrast, PlusXanthin demonstrates targeted action on the periocular area, with notable effects on fat reduction and pigmentation, supporting its use in anti-aging and eye contour treatments. These comparative results highlight the complementary profiles of extremophile-derived ingredients. Their distinct mechanisms and clinical outcomes support the development of multi-targeted cosmeceutical formulations, especially when combined in synergistic delivery systems. This diversity also reinforces the value of extremophiles as a sustainable reservoir of specialized bioactives for tailored skincare solutions. Beyond sourcing, sustainability is embedded in formulation strategies. For instance, upcycled phenolic extracts from lychee and dragonfruit peels—obtained via green solvents—exhibit strong antioxidant and anti-tyrosinase activity (92.3% inhibition), demonstrating how waste valorization can yield high-performance activities [144]. Likewise, alkyl glucosides, derived from coconut, corn, or potato, serve as biodegradable surfactants, replacing petrochemical emulsifiers in cleansers and shampoos [145]. Marine algae extracts, rich in fucoidan and phlorotannins, support anti-aging and collagen synthesis, with clinical improvements in dermal density (+40%) and wrinkle reduction (−35%). Their harvesting from renewable marine biomass aligns with blue bioeconomy principles [146]. Packaging innovations further reinforce sustainability. Unilever’s refill-reuse pilots and Lush Cosmetics’ naked products exemplify circular design, reducing virgin plastic use and promoting consumer engagement [147]. Emerging technologies include biodegradable active films, which release antioxidants or antimicrobials over time, enhancing product performance while minimizing waste. The concept of nutricosmetics, which emphasizes inside-out beauty, is gaining traction. Products combining topical and oral delivery systems, such as Imedeen^®^, Skinade^®^, and NutraFusion™, integrate marine collagen, vitamins, and plant extracts to deliver systemic and dermal benefits. These combined delivery platforms not only enhance efficacy but also reflect a holistic approach to wellness, aligning with consumer demand for integrative and sustainable skincare.

## 6. Conclusions

Extremophile-derived bioactives offer a unique combination of stability, multifunctionality, and sustainability, making them ideal for next-generation cosmeceuticals. This is the first comprehensive synthesis bridging extremophile biochemistry with skincare and nutraceutical perspectives. These bioactives, shaped by evolution in extreme environments, offer natural solutions for photoprotection, hydration, and oxidative stress mitigation. Beyond their biological advantages, sustainable production strategies—such as microbial fermentation and synthetic biology—are paving the way for scalable applications. Looking ahead, the development of personalized formulations and combined oral–topical approaches will be key to unlocking their full potential. Future efforts should focus on clinical trials, standardized efficacy markers, emerging omics tools for mechanism elucidation and sustainable manufacturing strategies to fully unlock the potential of extremophile-based skincare.

## Figures and Tables

**Table 1:** Correlation between environmental stress factors and extremophile-derived bioactive compounds with cosmetic relevance. Each stressor drives the synthesis of specific molecules that confer adaptive advantages in extreme habitats and multifunctional benefits in skincare, including UV protection, hydration, antioxidant defense, and anti-pollution effects. References indicate key studies supporting each stress–bioactive association.

Stress Factor	Bioactive Compound(s)	Function in Cosmetics
Radiation (UV, ionizing)	Mycosporine-like amino acids (MAAs), Scytonemin, Carotenoids (e.g., deinoxanthin), *Ectoine*	UV protection, antioxidant, anti-photoaging
High Salinity	*Ectoine*, Hydroxyectoine, Glycine betaine, Trehalose	Hydration, anti-inflammatory, barrier support
Cold (psychrophilic)	Antifreeze proteins (AFPs), Exopolysaccharides (EPSs), Trehalose	Moisturization, cryoprotection, anti-pollution
Heat (thermophilic)	Extremozymes (proteases, lipases, amylases), Chaperones	Enzymatic exfoliation, stability in formulations
Acidic pH	Organic antioxidants (e.g., glutathione), Peroxidases	Anti-oxidative, anti-inflammatory
Desiccation	*Ectoine*, Hydroxyectoine, EPSs	Moisturization, barrier protection
High Pressure	Pressure-stable extremozymes	Robust processing, formulation stability
Metal-rich environments	EPS with chelating properties	Anti-pollution, detoxifying claims

**Table 2:** Extremophilic microorganisms and their EPSs with cosmetic relevance.

Microorganism	EPS Name/Type	Key Properties
*Vreelandella titanicae* Zn11_249	Branched heteropolysaccharide	Antioxidant, non-cytotoxic, cosmetic potential
*Geobacillus*, *Parageobacillus*, *Aeribacillus*, *Anoxybacillus*	Thermophilic EPS	Antioxidant, antibacterial, prebiotic
*Pseudoalteromonas* sp. LP6-12-2	Mannose-rich EPS	Immunomodulatory, anti-aging potential
*Halomonas* sp. DT-Z4	Fructose-dominated EPS	High thermal stability, oil retention
*Alkalihalobacillus* sp.	EPS under alkaline stress	Biosorption, rheology modifier
*Bacillus* sp. NIOTSM16	EPS with fatty acids	Moisturizing, anti-pollution
*Rhodotorula glutinis* (cold-adapted yeast)	β-D-glucan EPS	Antioxidant, antibiofilm, cryoprotectant
*Chlamydomonas* sp. ASYA25	Microalgal EPS	Cryoprotectant, membrane integrity

**Table 3:** Limitation of extremophile-derived compounds for cosmeceutical and nutraceutical applications.

Compound Class	Limitations and Risks	References
*Ectoine/Hydroxyectoine*	Limited large-scale production due to cost; regulatory constraints in food use; potential allergenicity not fully evaluated.	[36,80,83]
Extremozymes	Expensive production and purification; possible immunogenicity if used in topical or ingestible formulations; few toxicity studies in humans.	[8,9]
Carotenoids	Instability under light and oxygen; bioavailability issues; production yield still limited for some extremophilic sources.	[85]
EPS	Complex purification and structure variability; high production cost.	[101]
PHA	High production costs of PHA compared with other (bio)polymers	[118]
MAAs	Low accumulation of MAAs in organisms, limited green extraction process, difficult identification, and high cost	[128]

## Data Availability

No new data were created or analyzed in this study.
